# Invasive fungal infections in liver diseases

**DOI:** 10.1097/HC9.0000000000000216

**Published:** 2023-08-28

**Authors:** Nicolas Barros, Russell E. Rosenblatt, Meaghan M. Phipps, Vladislav Fomin, Michael K. Mansour

**Affiliations:** 1 Department of Medicine, Indiana University School of Medicine, Indianapolis, Indiana, USA; 2 Division of Infectious Diseases, Department of Medicine, Indiana University Health, Indianapolis, Indiana, USA; 3 Department of Gastroenterology and Hepatology, Weill Cornell Medicine, New York, New York, USA; 4 Division of Digestive and Liver Diseases, Department of Medicine, Columbia University Irving Medical Center, New York, New York, USA; 5 Department of Gastroenterology and Hepatology, Weill Cornell Medicine, New York, New York, USA; 6 Department of Medicine, Harvard Medical School, Boston, Massachusetts, USA; 7 Division of Infectious Diseases, Department of Medicine, Massachusetts General Hospital, Boston, Massachusetts, USA

## Abstract

Patients with liver diseases, including decompensated cirrhosis, alcohol-associated hepatitis, and liver transplant recipients are at increased risk of acquiring invasive fungal infections (IFIs). These infections carry high morbidity and mortality. Multiple factors, including host immune dysfunction, barrier failures, malnutrition, and microbiome alterations, increase the risk of developing IFI. *Candida* remains the most common fungal pathogen causing IFI. However, other pathogens, including *Aspergillus, Cryptococcus, Pneumocystis*, and endemic mycoses, are being increasingly recognized. The diagnosis of IFIs can be ascertained by the direct observation or isolation of the pathogen (culture, histopathology, and cytopathology) or by detecting antigens, antibodies, or nucleic acid. Here, we provide an update on the epidemiology, pathogenesis, diagnosis, and management of IFI in patients with liver disease and liver transplantation.

## INTRODUCTION

Invasive fungal infection (IFI) is increasingly recognized as an emerging complication in patients with liver disease, including those with acute liver failure, severe alcohol-associated hepatitis (AH), decompensated cirrhosis, and those who undergo liver transplantation.^1^ IFIs are associated with increased morbidity and mortality. In patients with decompensated cirrhosis, IFI can occur in up to 10% of the patients admitted to the hospital, while, in liver transplant recipients, it can reach 40%.^2^

Host immune dysfunction, barrier failures, malnutrition, and microbiome alterations have been ascribed to increase the risk of developing IFI.^3^ *Candida* species remain the most common pathogen associated with IFI though the epidemiology is shifting with rising cases of other fungi, including *Cryptococcus*, and molds, such as *Aspergillus* spp.^1^ Despite the high mortality and relatively high infection rate, there is currently a lack of systematic literature evaluating the burden, risk factors, and mortality of IFI in patients with liver disease. Here, we provide an update on the epidemiology, pathogenesis, diagnosis, and management of IFI in liver disease.

### Epidemiology

#### IFIs in patients with cirrhosis

The risk of IFI in patients with liver disease is proportional to the severity of the liver disease. In compensated cirrhosis, the prevalence is low, especially when compared with bacterial infections; however, in patients with decompensated cirrhosis or those critically ill, the rates of IFI can be as high as 10%–14%.^1^ Furthermore, compared with other groups of patients with high acuity illness, such as intensive care unit (ICU)–requiring patients or those with severe cardiovascular disease, patients with cirrhosis are more likely to be colonized with fungi and develop IFI.^4,5^ About 25% of patients with cirrhosis admitted to the ICU are colonized with fungi.^4^

A meta-analysis of 31984 patients with cirrhosis showed that invasive candidiasis is the most common IFI, with a pooled prevalence ranging from 2% to 8%. *Candida albicans* was the most commonly isolated species though nonalbicans *Candida* species have increased during the past decade (8%–41%).^1^ Invasive aspergillosis was the second most common IFI, with a pooled prevalence ranging from 1.5% to 5.3%. The sites of the IFI included genitourinary (3.3%), bloodstream (2.1%), pulmonary (1.9%), peritoneum (0.7%), and central nervous system (0.5%).

Spontaneous peritonitis is common, occurring in 15%–26% of hospitalized patients with cirrhosis and ascites.^6^ In a study of 231 patients with spontaneous peritonitis with cirrhosis, 3.5% of the cases were found to be caused by fungal infections, specifically *Candida* species. *Cryptococcus neoformans*, although much rarer, can also cause spontaneous fungal peritonitis.^7^

#### IFIs in patients with AH

Limited studies have described the prevalence of IFI in patients with AH. In those studies, the prevalence of these infections ranged between 10% and 15%.^3^ A study evaluating 131 patients admitted to the hospital with severe hepatic failure due to AH showed that 14.5% had an IFI. Candidemia was present in 8.4% of the patients and invasive aspergillosis in 4.6%.^8^ In a small cohort of 32 patients admitted for management of AH, 10% of the patients developed candidemia within 1 month from admission.^9^ The high incidence of invasive candidiasis may be related to the abundance of *Candida* in the microbiome of those with AH, increased use of catheters, such as central venous and urinary catheters, and total parenteral nutrition.^10,11^

Another study investigating 94 patients with biopsy-proven AH showed that 16% had invasive aspergillosis, of which 87% were in the setting of corticosteroid use.^12^

In addition to increased risk for invasive aspergillosis and candidiasis, there has been recent evidence of *Pneumocystis jirovecii* pneumonia (PCP) in this patient population, with one study including 7 patients with biopsy-proven acute AH and PCP, 6 had received corticosteroids, and all of whom died ^13^

#### IFIs in liver transplant recipients

IFIs are more common among patients after liver transplants than those with decompensated cirrhosis. The incidence of IFI after liver transplantation ranges from 4% to 40%.^14–17^

*Candida* spp. remains the most common IFI post-liver transplantation representing up to 80% of the cases.^18,19^ However, the rates of infection vary with time from transplantation. Invasive candidiasis represents up to 85% of the cases in the first 6 months following transplantation but drops to 40% in the subsequent 6 months.^20^ *C. albicans* is the most commonly isolated pathogen ranging from 45% to 65% of infections. However, recent data show a change in *Candida* species over time, with a rise in nonalbicans species, including *C. parapsilosis,* responsible for 19%–28% of all invasive candidiasis.^21^

Invasive aspergillosis occurs in 2% of liver transplant recipients, with most cases resulting from pulmonary infections.^22,23^ The Swiss Transplant Cohort Study showed that the median time between transplantation and invasive aspergillosis is 18 days (IQR 9–122) though prior studies showed that most cases occurred after 90 days.^14,24,25^ Patients with pretransplant colonization with *Aspergillus* spp. can still undergo liver transplantation if they are suitable candidates and receive appropriate antifungal therapy.^26^

Among patients with decompensated cirrhosis and liver transplant recipients, *Cryptococcus* remains the third most common cause of fungal infection.^27,28^ Cryptococcal infection may have a worse prognosis in patients with liver diseases owing to a delay in diagnosis. The most common manifestations of cryptococcal infection include pneumonia, meningitis, peritonitis, cutaneous infections, and cryptococcemia.^29^ Decompensated cirrhosis (Child-Pugh Class B or C) had more frequent extrapulmonary manifestations of cryptococcal disease.^30^

While cryptococcal infections after transplant are more commonly due to a *de novo* infection, prior cryptococcal infections and even rare cases of donor-derived infections must be considered.^31–33^

The time of onset for a cryptococcal infection is earlier (<12 mo) among liver transplant recipients compared with other solid organ transplants, but, for infections that occur after 2 years, the central nervous system infection is more likely.^34^ In addition, liver transplant recipients, as opposed to different types of solid organ transplant recipients, had a 6-fold higher risk of developing disseminated disease.^35^

PCP is described in all solid organ transplant recipients. Among liver transplant recipients, rates have decreased with the introduction of widespread prophylaxis and reduced intensity of immunosuppression regimens. Recent studies have struggled to describe PCP incidence in an era with now relatively widespread prophylaxis. In a single-center cohort, rates of PCP were low at 0.8%, and of the cases of PCP identified, only 1 occurred within 6 months after transplantation when prophylaxis is typically recommended.^36^ However, in a cohort evaluating the rates of PCP in an era of routine prophylaxis, no cases of PCP were detected in patients taking prophylaxis during the first 6 months. In comparison, 9.5% of the liver transplant recipients who did not receive prophylaxis developed PCP.^37^

Donor-derived fungal infections can occur from direct transmission of donor infection to the recipient or, more commonly, from preservation fluid contamination or graft contamination during organ procurement. Contamination of the preservation fluid with *Candida* species occurs in 0.6%–4.1% of all preservation fluids, and *Candida albicans* represents 25%–64% of all *Candida* isolates. The contamination of preservation fluid increases the risk for vascular complications, including arteritis and aneurysms, surgical site infections, and graft loss.^38–41^ In a recent study, 28.6% of patients with contamination of preservation fluid with *Candida* species developed complications related to the infection with a 1-year mortality rate of 62.5%.^38^

Consensus guidelines from the European Association for the Study of the Liver (EASL) suggest that donors with isolated fungal infections are routinely used with acceptable outcomes though a course of antifungal therapy is usually warranted.^42^

### Risk factors

#### Risk factors in patients with decompensated cirrhosis

**FIGURE 1:**
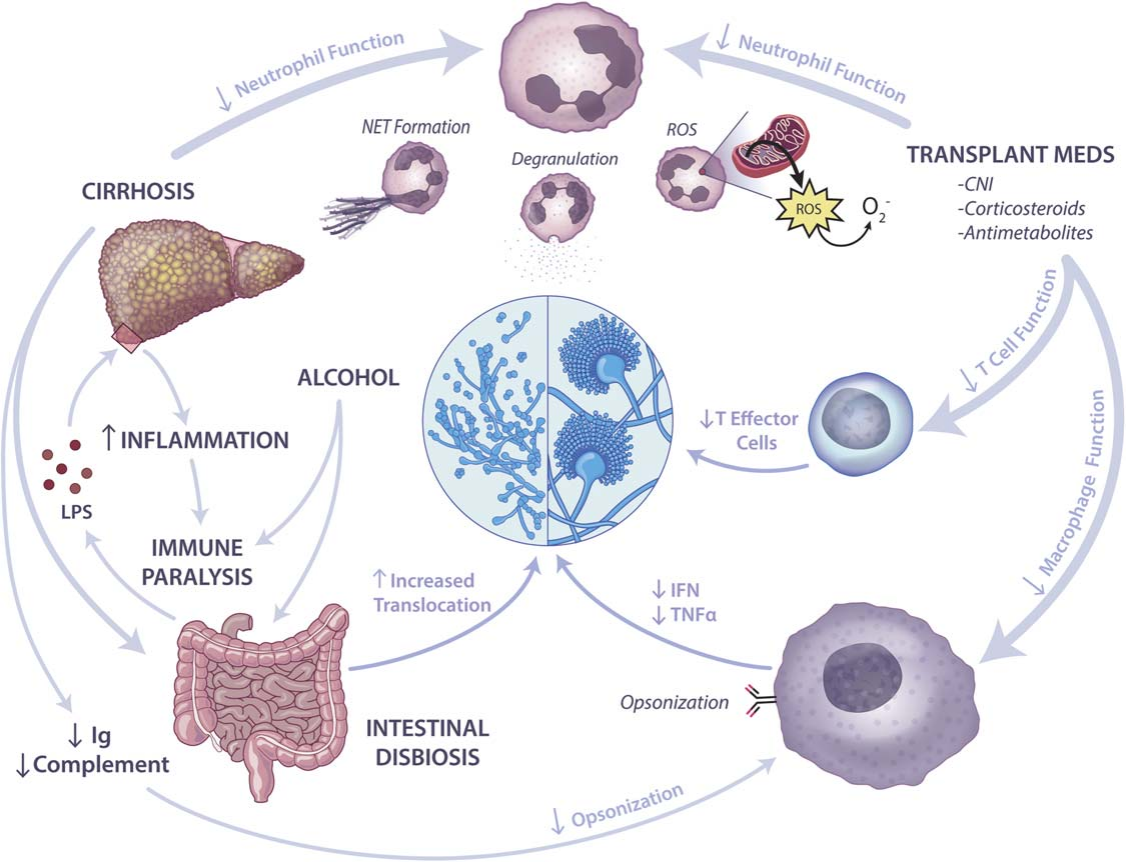
Immune dysfunction and invasive fungal infections in liver disease. Alcohol use leads to immune paralysis and increase gut permeability, which, in turn, leads to increased LPS in the circulation. LPS in the cirrhotic liver leads to increased inflammation leading to further immune paralysis. The decreased immunoglobulin and complement production from cirrhotic livers leads to decreased opsonization by human macrophages. In addition, the neutrophil function is decreased in cirrhosis. Transplant medications, including calcineurin inhibitors, corticosteroids, and antimetabolites, decrease neutrophil, macrophage, and T-cell function. Abbreviations: CNI, calcineurin inhibitor; IFN, interferon; LPS, lipopolysaccharide; NET, neutrophil extracellular trap; ROS, reactive oxygen species. Illustration by Nicole Wolf, MS, ©2022.

Patients with cirrhosis are at increased risk of infection due to immune dysfunction, which is multifactorial in etiology (Figure 1). There are liver disease-specific causes (such as decreased T-cell function in individuals with high alcohol consumption and altered immunoglobulin production), impaired neutrophil and monocyte activity, and poor opsonization (notably in the ascites fluid) and iron overload.^18,28,43^ (Table 1) In addition, as liver disease progresses, increased systemic inflammation can lead to functional paralysis of immune cells. Intestinal dysbiosis, including the overgrowth of *Candida* species, leads to intestinal barrier disruption and translocation of bacteria, fungi, and biologically active microbial ligands.^19^ Finally, the patient with decompensated cirrhosis is often in a pre-existing weakened state due to malnutrition and sarcopenia.

**TABLE 1:** Risk factors, types of infections and most common agent of invasive fungal infections

	Risk factors	Type of infections	Most common agent
**Decompensated cirrhosis**			
	Immune paralysis	Urinary tract	*Candida spp*
	Intestinal dysbiosis	Fungemia	*Aspergillus spp*
	Malnutrition	Pulmonary	*Cryptococcus neoformans*
	High MELD score	Peritonitis	*Pneumocystis jirovecii*
	Prolonged hospitalization		
**Alcohol related hepatitis**			
	ICU stay	Fungemia	*Candida spp*
	Decreased T cell function	Pulmonary	*Aspergillus spp*
	Decreased neutrophil and monocyte activity		*Pneumocystis jirovecii*
	Increased IL-10		
	Steroid use		
	Prolonged hospitalization		
**Liver transplant**			
	Prolonged or repeat operation	Fungemia	*Candida spp*
	Renal failure	Deep surgical site infection	*Aspergillus spp*
	High transfusion requirements	Peritonitis	*Cryptococcus neoformans*
	Coledocohehunostomy	Pulmonary	Dimorphic fungi
	Perioperative colonization with fungal species		*Pneumocystis jirovecii*
	Immunosuppression		

Abbreviations: ICU, intensive care unit; MELD, Model for End-Stage Liver Disease.

Unsurprisingly, the most common risk factors for acquiring fungal infections are critical illness and the severity of liver disease.^20,44^ Prolonged hospitalization and the need for ICU-level care are strong risk factors associated with developing IFI. In a North American Consortium for the Study of End-Stage Liver Disease (NACSELD) study, all fungal infections were nosocomial. In addition, antibiotic exposure, central venous catheter use, and total parenteral nutrition have been associated with an increased risk of IFI.^45^ While these are likely independent risk factors for IFI, they are also markers of critical illness, and the exact cause-and-effect relationships remain unclear.^1,46–48^ The addition of comorbidities, such as diabetes, has also been associated with IFI.^1,45^

The severity of liver disease, specifically acute-on-chronic liver failure (ACLF), generally defined as an acute decompensation in a patient with chronic liver disease, has been identified in several studies as a risk factor for fungal infections.^1,47,49^ IFIs have been cited as the cause of ACLF and/or a resulting complication of existing ACLF.^45,49^ Similarly, higher Model for End-stage Liver Disease (MELD) scores and complications, including hepatorenal syndrome, have also been implicated as risk factors for IFI among patients with cirrhosis.^48^

#### Risk factors in patients with alcohol-associated liver disease

Patients with alcohol-associated liver disease, including those without cirrhosis but with severe acute AH, are at increased risk for IFIs. While many patients with severe AH have underlying cirrhosis, there are unique and additional mechanisms that predispose patients with severe AH to infection. A prospective study of patients with severe AH, compared with those with stable and advanced alcohol-associated cirrhosis, demonstrated that patients with severe AH exhibited a more immunosuppressive and IL-10-dominated state, leading to reduced antimicrobial immunity. These changes existed before the addition of steroids, often administered in patients with severe AH, leading to further immunosuppression and a higher risk of infection.^50^

#### Risk factors in liver transplant recipients

IFIs are more common among patients after liver transplantation than in patients with decompensated cirrhosis, largely due to the intentionally increased state of immunosuppression, though intrinsic neutrophil dysfunction has been previously reported.^51^ Liver transplant patients with IFI have poor outcomes, with as high as 66% mortality.^52^ However, various preoperative and perioperative risk factors are associated with increased risk for IFI after liver transplantation.

*Candida* colonization before liver transplantation increases the likelihood of invasive *Candida* infection following transplantation. In one retrospective study of over 500 patients, pretransplant *Candida* colonization was the only factor in multivariable analysis that increased the risk for IFI.^53^ The need for repeat laparotomy and retransplantation has also been shown in several studies to increase the risk for IFI. Other factors associated with increased risk for IFI include renal replacement therapy, high intraoperative blood product administration, and choledochojejunostomy.^54–58^

High pretransplant MELD scores have also been associated with an increased risk for IFIs. In a multinational study, 83% of patients with candidemia after liver transplantation had a pretransplant MELD score of >15.^59^ In a retrospective study, an MELD score of 20–30 was associated with a 2.1-fold increase in relative risk of IFIs, while scores ≥30 were associated with a 3.1-fold increase.^60^

A predictive model for risk stratification based on a retrospective analysis using creatinine >3.0 mg/dL, operative time of at least 11 hours, retransplantation, and early colonization showed that only 1% of patients with no risk factors developed IFI versus 67% of those with two or more risk factors.^56^ As a result, the American Society of Transplantation Infectious Diseases Community of Practice (AST IDCOP) has recognized patients as high risk for the development of invasive fungal disease if they have ≥2 of the following risk factors: prolonged or repeat operation, retransplantation; renal failure; high transfusion requirement (≥40 units of cellular blood products, including platelets, packed red blood cells, and autotransfusion); choledochojejunostomy; and *Candida* colonization in the perioperative period.^61^

### Diagnosis of IFIs

#### Candida

Invasive candidiasis encompasses bloodstream infections (candidemia), and those infections found beneath the mucosal surface or “deep-seated infections,” which can occur concurrently or independently. Isolation of *Candida* species using culture-based methods remains the gold standard for diagnosis though the sensitivity of this method varies between the type of infection and fungal load.^62^ The sensitivity of blood cultures with automated blood culture systems for bloodstream infections with or without deep-seated infections can reach up to 75%.^62^ However, the sensitivity is much lower in cases of intermittent candidemia and deep-seated candidiasis without candidemia. In those cases, the sensitivity falls between 21% and 71% with an overall sensitivity of about 50%.^63^ While the median turnaround time for blood and tissue cultures is 2–3 days, the identification at the species level using traditional techniques may take much longer.^64^ Culture-based diagnostic molecular assays, including matrix-assisted laser desorption/ionization-time of flight mass spectroscopy, decreased that time. However, they still require the isolation of pathogens using a solid culture media.^65^

Other molecular diagnostics tests can assess the species directly from the blood culture sample rather than waiting for isolation on solid media resulting in decreased time to identification at the species level. The FilmArray blood culture identification panel (BioFire Diagnostics LLC, Salt Lake City, UT) can identify 24 etiologic agents of sepsis, including 6 *Candida* species (*C. albicans*, *C. glabrata*, *C. krusei*, *C. parapsilosis*, *C. auris*, and *C. tropicalis*). The sensitivity and specificity for identifying these pathogens are 97.1%–99.9% and 99.9%–100%, accordingly.^66^ Another platform, the peptide nucleic acid fluorescent *in situ* hybridization (PNA FISH) assay, was first described in 2002 and is designed to identify *C. albicans* and *C. glabrata* with sensitivities of 98.7% and 100%, respectively, and 100% specificity for both components.^67^ The rapid identification of *C. albicans* can significantly reduce the use of echinocandins, resulting in appropriate antifungal stewardship and decreased costs.^68^

Culture-independent diagnostic tests, including 1–3 β-D glucan, a common component of the fungal cell wall, may be helpful to guide treatment decisions and antifungal stewardship. In 2 recent meta-analyses, the sensitivity of a positive result was 75%–80%, and the specificity was ~80%.^69,70^ While the positive predictive value is low even in populations with a high prevalence of candidemia, the negative predictive value remained excellent (>97%). Several studies of ICU patients at risk for candidemia have shown that antifungal therapy can be discontinued safely in patients with negative 1-3 β-D glucan.^71,72^

#### Cryptococcus

An important task during the diagnosis of cryptococcosis is to determine the severity and extent of infection and the site of infection (ie, isolated pulmonary cryptococcosis, cryptococcemia, cryptococcal meningitis, and so on) as it impacts both the diagnostic performance of tests and treatment (both antifungal of choice and duration of therapy).^73–75^

The most common radiographic finding is single or multiple pulmonary nodules though other findings include cavitary lesions, lobar consolidations, and mass-like lesions. Brain tissue is rich in substrates for phenol oxidase action, which may at least in part explain the tropism of *Cryptococcus* for the central nervous system (CNS).^32,74^

The diagnosis of cryptococcosis can be achieved by direct visualization of the yeast on histopathology, isolation through culture-based methods, detection of fungal antigens or antibodies, or through molecular tests (eg, nucleic acid amplification or hybridization). Isolation of *Cryptococcus neoformans* though culture remains the gold standard for diagnosing cryptococcal infection though typically takes 3–7 days. Cultures can be obtained from any biologic samples, including cerebrospinal fluid (CSF), sputum, blood, skin, and biopsies.

Sampling from CSF through lumbar puncture should be pursued in immunocompromised patients to exclude CNS infection. Increased intracranial pressure is a common feature of CNS cryptococcosis and has implications in the management. Hence, its measurement should be recorded in all cases. Direct visualization of the yeast in CSF using India ink staining is an inexpensive technique that used to be the most widely used but has now been displaced by detecting cryptococcal antigens. This shift from India ink was due to its low sensitivity (<86%) and being operator-dependent.^76^ The cryptococcal antigen can be detected through latex agglutination or lateral flow assay in different fluids, including serum, plasma, and CSF. The point-of-care lateral flow assay is less labor-intensive and is more sensitive. Currently, the lateral flow assay is the most commonly used diagnostic method to detect cryptococcal antigens with high sensitivity (93%–100%) and specificity (93%–98%) in both blood and CSF.^77,78^ Of note, false-positive serum cryptococcal antigen can occur in the setting of infection due to the fungus *Trichosporon asahii*.^79^

Nucleic acid amplification testing is an important diagnostic modality. There are several multiplexed assays that include *Cryptococcus*. Both the AusDiagnostic CSF panels (AusDiagnostics, Mascot, and Australia) and BioFire meningitis/encephalitis panel (bioMerieux, Marcy l’Etoile, France) can be used in CSF with a sensitivity of ~84%. Of note, there are several reports of negative BioFire meningitis/encephalitis panel (bioMerieux) in patients with culture-positive cryptococcal meningitis.^80,81^ These panels are unable to discriminate between *C. neoformans* and *C. gattii*.^82^

#### Aspergillus

Aspergillosis has 3 significant forms: invasive aspergillosis, chronic aspergillosis, and allergic bronchopulmonary aspergillosis. Here, we will describe the diagnosis of invasive aspergillosis as it is the most frequent form in patients with cirrhosis and liver transplant recipients.^1,83^

Pulmonary infection is the most common presentation, followed by invasive sinusitis, though dissemination and infection of other organs are well described.^84^ *Aspergillus* spp. is a frequent colonizer of the respiratory airways and can be isolated without evidence of invasive disease. Discerning colonization from invasive infection can be challenging. Therefore, the European Organization for Research and Treatment of Cancer and the Mycoses Study Group have developed consensus definitions of IFIs.^85^

Radiographic findings include consolidation-or-mass, halo signs (ground-glass attenuation surrounding a pulmonary nodule-or-mass), and lesions suggestive of angio-invasiveness (presence of at least 2 of the 3 following findings: a halo sign, an infarct-shaped consolidation, an internal low attenuation, cavity, or air-crescent sign). While a halo, cavity, or air-crescent sign is commonly seen in neutropenic patients (78%, 55%, and 60%, respectively), they are seldomly found in non-neutropenic patients (56%, 26%, and 32%, respectively).^86^ In liver transplant recipients, multiple nodules with ground-glass opacities are the most common features (80%).^87,88^

Since radiographic changes can be nonspecific, microscopy and culture of respiratory samples [sputum, tracheal aspiration, and bronchoalveolar lavage (BAL) fluid] remain the cornerstone for microbiological confirmation. However, respiratory culture sensitivity is low, with only 50%–64% positivity.^89,90^

The *Aspergillus* cell wall contains multiple polysaccharides, including galactomannan and glucans, which can be used as nonculture diagnostic methods. *Aspergillus* galactomannan assay can be performed in serum or BAL.^84^ Circulating serum galactomannan correlates with the invasive growth of *Aspergillus* species. In patients with hematological malignancies and stem cell transplant recipients, the fungal burden and angio-invasive disease tend to be high, which correlates with a high sensitivity of the test (>70%).^91^ However, studies involving solid organ transplant recipients (including liver transplants) show an overall sensitivity of only 22%. The specificity remained high across the different study groups (~85%). Using an index cutoff of 0.5 instead of 1 increases the sensitivity of the test but decreases its specificity.^89^ Given the overall poor performance of serum galactomannan in patients without hematological malignancies or stem cell transplantation, using galactomannan for diagnosing invasive aspergillosis in patients with liver disease is not recommended.^92^

The current Infectious Diseases Society of America guidelines recommend a bronchoscopy with bronchoalveolar lavage in patients suspected of invasive pulmonary aspergillosis without contraindications.^84^ Detection of *Aspergillus* galactomannan in the bronchoalveolar fluid has been shown to have a sensitivity of 84%–86% and a specificity of 89%.^93,94^ Of note, *Aspergillus* galactomannan can be false positive in patients with other invasive mycoses, including histoplasmosis.^95^ Just as in diagnosing invasive candidiasis, using 1-3 β-D glucan is limited for invasive aspergillosis due to its low specificity.^96^

#### Pneumocystis

*Pneumocystis jirovecii* infection occurs almost exclusively in the pulmonary parenchyma, with extrapulmonary infections being exceedingly rare. The classic radiographic findings include bilateral perihilar interstitial infiltrates and ground-glass opacification. Less frequent findings include pulmonary cysts, pneumothoraces, nodules, and lobar consolidations.^97^

*Pneumocystis jirovecii* is unable to grow or replicate *in vitro*. Hence, cultures cannot be used to diagnose PCP. Identification of the pathogen in respiratory samples through direct immunofluorescence antibody stains and molecular testing is the most common technique used to diagnose PCP. Bronchoalveolar lavage fluid is considered to be the highest quality respiratory sample.^98^ The sensitivity and specificity of direct immunofluorescent antibody staining from bronchoalveolar lavage samples are 91%–93% and 95%–100%, respectively.^99,100^ NAAT has been shown to be more sensitive than immunofluorescent staining with a pooled sensitivity of 97%–99% and a pooled specificity of 90%–94%.^101^

Serum 1-3 β-D glucan is a highly sensitive test with a sensitivity of around 95%. However, the specificity is only 75%. The test has an excellent negative predictive value (>95%) rendering PCP unlikely if a negative test is obtained.^101^

### Dimorphic fungi

Dimorphic fungi encompass many organisms that grow as molds in the environment and as yeast in humans. These fungi have well-described endemicity regions, which has prompted their name of endemic mycoses. The more commonly occurring dimorphic fungi infections in the United States are histoplasmosis, blastomycosis, and coccidioidomycosis.^102^ While the infection is usually mildly symptomatic and self-resolved in healthy individuals, patients with impaired immunity, including individuals with liver disease and transplantation, can develop severe disease with dissemination.^103,104^

In the United States, histoplasmosis and blastomycosis have a distinct geographic distribution. The highest endemicity areas are in the Ohio and Mississippi Rivers Valleys, upper Midwest, and US states and Canadian provinces bordering the Great Lakes. In addition, histoplasmosis has been identified in all continents except for Antarctica.^102^ Coccidioidomycosis thrives in arid climates with low annual rainfall and alkaline soils. Most infections occur in the Southwest United States and areas of Mexico adjacent to the US border. Some regions of Central America and South America are mildly endemic.^102^

Most infections present with pulmonary involvement. Imaging of the lungs may show a myriad of presentations, including pulmonary nodules, cavitations, and consolidations.^103^ However, with decreased cell-mediated immune responses, dissemination into any organ can occur. Clinical findings in patients with disseminated disease can include fever, chills, night sweats, weight loss, hepatosplenomegaly, pancytopenia, hepatic enzyme derangements, and gastrointestinal involvement.^34^

While the definitive diagnosis is made by direct visualization of yeast forms or growth of the pathogen from tissue samples, frequently positive cultures may require over 2 weeks of incubation. Detection of either fungal antigens and/or antibodies is useful for the diagnosis of these infections.^34^

#### Histoplasma

Detecting the *Histoplasma* polysaccharide galactomannan antigen by enzyme immunoassay (EIA) in human samples indicates infection. Human samples may include serum/plasma, urine, BAL, and (CSF. Urine *Histoplasma* antigen testing has an overall sensitivity of 92%.^35^ However, in patients with disseminated disease, the sensitivity increases to 97% compared with only 73% for those with localized pulmonary disease. Serum/plasma testing has a slightly lower sensitivity (about 86%), while BAL has a sensitivity of up to 93%.^105^ The specificity of *Histoplasma* antigen detection is above 90%, irrespective of the sample type.

The galactomannan antigen detected in histoplasmosis is similar to the blastomycosis galactomannan antigen leading to cross-reactivity. Cross-reactivity with other IFIs, such as *Paracoccidioides* spp., *Sporothrix schenckii*, *Coccidioides* spp., *Aspergillus* spp., and *Talaromyces marneffei*, can occur. Comparing the concentrations may help differentiate between different infections as the offending pathogen usually has the highest detectable concentration.^34,105–109^

Detection of antibodies against *Histoplasma* (either by immunodiffusion or complement fixation) is less useful as immunocompromised patients may not mount an appropriate response and may have negative results despite active infection. In a cohort of 152 solid organ transplant recipients with histoplasmosis, only 36% had a positive *Histoplasma* antibody test (pulmonary disease: 62% and disseminated disease: 28%).^110^ In addition, *Histoplasma* antibodies may be detected in up to 10% of the population in highly endemic areas, though usually at low titers.^111^

#### Blastomyces

The definitive diagnosis of blastomycosis can be achieved by isolation of *Blastomyces* spp. in tissue samples or body fluids. Detection of the *Blastomyces* polysaccharide galactomannan antigen by EIA in urine has a sensitivity that varies from 76% to 90%. In serum, the sensitivity is slightly lower ranging from 56% to 82%.^112^

Reliable antibody testing has remained elusive. An EIA targeting BAD-1 surface protein provided a sensitivity and specificity of 88% and 99%, respectively, though larger studies are needed to confirm this performance.^113^

### Coccidioides

Serologic testing can be useful in the detection of coccidioidomycosis. Currently, there are 3 serologic assays, including EIA, immunodiffusion-based assay, and complement fixation assay. The EIA appears to have the highest sensitivity (83%–100%) though it may be limited by increased false-positive IgM. Both immunodiffusion-based and complement fixation assays have a sensitivity of 60%–70% with a specificity of up to 98%. The detection of *Coccidioides* antigen by EIA in urine or serum/plasma may be helpful in immunocompromised patients with sensitivities of 50%–71% and 28%–73%, respectively, and specificities of above 90%.^78^

### Management and prevention of IFIs

#### Candida

Empiric therapy should be started as soon as the diagnosis is suspected, as early treatment initiation decreases mortality.^114^ Echinocandins are recommended as the first line of therapy for candidemia by the Infectious Diseases Society of America except for the management of infections of tissue with low penetration, including eyes, CNS, and urinary tract.^115^ (Table 2) There is no benefit with any of the specific echinocandins (eg, micafungin, caspofungin, and anidulafungin) over others, and for now, they are considered interchangeable.^116^ The use of novel long-acting echinocandins, such as rezafungin, will require additional investigation. Higher than recommended dosing is not shown to be superior to standard dosing for *Candida* spp.^117^ Amphotericin has similar efficacy to echinocandins but more side effects, yet it remains an alternative if echinocandins are not available or are contraindicated.^118^ Fluconazole can be considered an acceptable alternative as initial therapy in patients who are not critically ill and unlikely to be infected with a fluconazole-resistant *Candida* species.^115^

**TABLE 2:** Treatment of most common invasive fungal infection in patient with liver disease

Type of infection	Primay	Alternative	Duration	Comments
**Candidemia and invasive candidiasis**	Echinocandin: Micafungin 100 mg/d Caspofungin 70 mg loading then 50 mg/d Anidulafungin 200 mg loading then 100 mg/d	Azole: Fluconazole 12 mg/kg loading dose and then 6 mg/kg IV/PO daily if the patient is not critically ill and fluconazole-resistance is unlikely. Liposomal amphotericin B (3–5 mg/kg IV daily) is a reasonable alternative if other antifungals are not an option (availability or resistance).	If there is no obvious metastatic complication, therapy should be continued for 2 weeks from documented clearance of bloodstream infection.Transition to an azole is recommended after 5–7 days if the patient is stable and the isolates are susceptible to the selected azole.	CVCs should be removed as early as possible. Dilated ophthalmological examination should be performed in all patient with vision changes or in those who are unable to provide this information. For invasive candidiasis, duration of therapy may be at least 2 weeks and resolution of symptoms attributable to invasive candidiasis.Source control, with drainage and/or debridement should be included in intra-abdominal candidiasis or abscesses/collections.
**Invasive pulmonary aspergillosis**	Voriconazole 4 mg/kg PO (or IV) for 2 doses followed by voriconazole 2 mg/kg PO (or IV) every 12 hours	Azole: Isavuconazole 372 mg PO (or IV) every 8 hours for 6 doses followed by isavuconazole 372 mg PO (or IV) daily Posaconazole 300 mg IV every 12 hours for 2 doses followed by 300 mg IV daily Delayed release Posaconazole 300 mg PO every 12 hours for 2 doses followed by 300 mg PO daily Oral suspension 200 mg PO every 8 hours Liposomal amphotericin B (3–5 mg/kg IV daily) is a reasonable alternative if other antifungals are not an option (availability or resistance).	12 weeks and resolution of symptoms and radiographic findings.	Drug-drug interactions are very important with the use of these azoles. Needs to be discussed with pharmacy as other medications may need to be adjusted. Azoles have the risk of prolonged QTc (except isavuconazole). Hepatotoxicity can occur with these drugs. Therapeutic drug monitoring is important for azoles (except for fluconazole and isavuconazole).
**Cryptococcosis** Localized pulmonary infection Disseminated/CNS cryptococcus infection	Fluconazole 400 mg PO every 24 hours Induction (2 weeks): Liposomal Amphotericin B (3–5 mg/kg IV daily) + Flucytosine 25 mg/kg PO 4 times daily+ flucytosine 25 mg/kg PO 4 times daily Consolidation (8 weeks)Fluconazole 800 mg PO every 24 hours Maintenance (42 weeks)Fluconazole 400 mg PO every 24 hours	Induction: Single-dose lipid formulation amphotericin B (AmB) 10 mg/kg + flucytosine 25 mg/kg PO 4 times daily + fluconazole 1200 mg PO/d Consolidation (8 weeks)Fluconazole 800 mg PO every 24 hours Maintenance (42 weeks) Fluconazole 400 mg PO every 24 hours	6–12 months 12 months	Management of intracranial hypertension with recurrent lumbar punctures is associated with improved outcomes. Flucytosine therapeutic drug monitoring is required after 3–5 days of therapy.
**Pneumocystosis**	TMP-SMX 15–20 mg/kg of TMP component PO (or IV) divided every 6 or 8 hours for 21 days OR Dapsne 100 mg PO daily + TMP 5 mg/kg every 8 hours for 21 days*** if while on room air the patient has pO_2_ <70 mmHg or alveolar-arterial (A-a) oxygen gradient ≥ 35 mmHg, or hypoxemia on pulse oxymetry then adjuvanct glucocorticosteroid therapy is indicated: Prednisone 40 mg PO every 12 hours for 5 days followed by prednisone 40 mg PO every 24 hours for 5 days followed by prednisone 20 mg PO daily for 11 days	Clindamycin 600 mg IV every 8 hours + primaquine base 15-30 mg PO every 24 hours for 21 days + prednisone 40 mg PO every 12 hours for 5 days followed by prednisone 40 mg PO every 24 hours for 5 days followed by prednisone 20 mg PO daily for 11 days OR pentamidine 4 mg/kg IV every 24 hours for 21 days + prednisone 40 mg PO every 12 hours for 5 days followed by prednisone 40 mg PO every 24 hours for 5 days followed by prednisone 20 mg PO daily for 11 days	21 days	Lifelong secondary prophylaxis is required on patients with persistent immunosuppression.

Abbreviations: CNS, central nervous system; CVC, Central venous catheters; QTc, corrected QT interval.

Initial echinocandin treatment can be followed within 3–5 days by step-down to oral triazole therapy depending on the *Candida* isolated and susceptibility results.^119^ Removal of central line catheters and drainage of the potential source impact survival.^120^ Therapy for candidemia is usually continued for 2 weeks from documented clearance of bloodstream infection. However, longer durations may be required in patients with metastatic complications (eg, osteomyelitis). Infectious diseases consultation is associated with significantly lower mortality.^121^

The necessity of echocardiography in patients with candidemia remains unclear, and there is no consensus among professional organizations. In a large retrospective study of 1873 patients with candidemia, Foong et al^122^ showed that 2.5% of patients with candidemia had infective endocarditis. The Infectious Diseases Society of America recommends an echocardiography in patients with persistent candidemia despite appropriate therapy, while the European Society of Clinical Microbiology and Infectious Diseases guidelines recommend an echocardiography in all patients with candidemia.^115,123^

Ocular candidiasis is a feared complication of candidemia. The prevalence of endophthalmitis in patients with candidemia is reported to range from 0% to 5%.^124,125^ In a systematic review of 7472 patients who underwent ophthalmologic screening for candidemia, Breazzano and colleagues described that only 0.9% of the patients had concordant endophthalmitis (defined as candida chorioretinitis with the extension of the surrounding inflammation into the vitreous or vitreous abscess manifesting as intravitreal fluff balls). They concluded that inconsistent definitions of endophthalmitis may lead to discrepancies in its incidence.^126^ In a recent meta-analysis of 70 studies involving 8109 patients, Phongkhun et al^127^. showed that the prevalence of concordant endophthalmitis was between 1.3% and 2.6%. There is no consensus on whether ocular examination should be performed in all patients with candidemia or only on those with symptoms. The Infectious Diseases Society of America recommends a dilated fundoscopic examination in all non-neutropenic patients with candidemia to assess for endophthalmitis.^115^ The American Academy of Ophthalmology recommends a fundoscopic examination only in symptomatic patients.^128^

Noninvasive presentations, such as esophageal candidiasis, are frequent in patients with cirrhosis and liver transplant recipients. The independent predictors for developing esophageal candidiasis include alcohol binge, HCC, upper gastrointestinal bleeding, ACLF, diabetes, and higher MELD score.^129^ The diagnosis should be suspected in patients with odynophagia. While the confirmatory diagnosis requires an upper endoscopy with or without biopsies, we recommend starting therapy (particularly on patients with thrush) and proceeding to endoscopy if there is a lack of improvement within 72 hours. Since almost all infections are due to *C. albicans*, fluconazole is the agent of choice and should be continued for 14–21 days. In contrast to esophageal candidiasis, where systemic therapy is recommended, oropharyngeal candidiasis can be managed with topical therapy (eg, nystatin suspension), and the duration can be shorter (7–14 d).^115^

Given the high morbidity and mortality associated with IFI, multiple studies have addressed the use of prophylactic antifungals. Prophylaxis against invasive candidiasis should only be targeted to high-risk populations. The American Society of Transplantation Infectious Diseases Community of Practice (AST IDCOP) has consensus guidelines about patient selection for *Candida* prophylaxis, and they recommend prophylaxis for high-risk patients.^61^ While prophylaxis has been demonstrated to reduce IFI, it has not been shown to reduce overall post-transplant mortality. Many studies use fluconazole for invasive candidiasis prophylaxis given the relatively low cost, oral option, and overall good proven efficacy.^2,130^

The AST IDCOP guidelines also recommend prophylaxis in patients with *Candida* colonization in the perioperative period (isolation of *Candida* in at least 2 nonsterile sites in the 4 weeks preceding transplantation). However, screening for colonization is not often assessed.

In a randomized controlled trial, liver transplant recipients who met at least 2 criteria for high risk of IFI received either liposomal amphotericin B or fluconazole for 14 days. Fourteen percent of patients developed IFI, almost all with *Candida* species, with similar rates in both arms. No deaths were attributed to the IFI prophylaxis or IFI itself.^131^ Another meta-analysis of 14 trials demonstrates a reduction of IFI in antifungal prophylaxis use but again did not impact all-cause mortality.^132^ While there was no mortality difference, we recommend prophylactic antifungal use in patients who meet high-risk criteria in the AST IDCOP guidelines.

The AST IDCOP recommends that antifungal prophylaxis should be given to all adult liver transplant recipients at high risk for the development of invasive candidiasis (≥2 of the following risk factors, prolonged or repeat operation, retransplantation, renal failure, high transfusion requirement (transfusion of ≥40 units of cellular blood products including platelets, packed red blood cells, and autotransfusion), choledochojejunostomy, and *Candida* colonization in the perioperative period.^61^

The duration of prophylaxis is poorly defined, with reports ranging from 5 days up to 8 weeks. However, most studies recommend up to 4 weeks of prophylaxis or when the patient is discharged.^15,58,131,133–137^

#### Cryptococcus

Patients with severe or disseminated disease and or meningitis should be treated aggressively. Most of the studies have been performed in HIV-infected individuals and have been extrapolated to other populations. The treatment includes 3 phases: induction, consolidation, and maintenance.^74^ The Infectious Diseases Society of America states that the induction phase should include 2 weeks of liposomal amphotericin B 3 mg/kg and 5-flucytosine 25 mg/kg PO every 6 hours.

The ACTA study showed that 1 week of amphotericin B and flucytosine was effective for induction.^138^ More recently, the Ambition study group showed that a single-dose liposomal amphotericin B (10 mg/kg) with 14 days of flucytosine (25 mg/kg every 6 h) and fluconazole (1200 mg/d) was not inferior to amphotericin B (deoxycholate) with flucytosine for 7 days and was associated with fewer side effects. Of note, most of the participants were young and did not have known liver disease.^139^ Fluconazole (like all azoles) has significantly associated hepatotoxicity, which is dose dependent.^140^ It is unknown if patients with severe liver disease would be able to tolerate a daily dose of 1200 mg of fluconazole for 14 days.

Consolidation therapy includes high-dose fluconazole (400–800 mg/d) for 8 weeks. Once consolidation therapy is complete, patients can be transitioned to maintenance therapy with fluconazole (200-400 mg daily) for at least 12 months.^74,141^

Intracranial hypertension occurs in 50%–70% of patients with cryptococcal meningitis.^142^ Management of it is pivotal and has been associated with the reduction of mortality.^143^ Daily lumbar punctures may be required to normalize the intracranial pressure.

Patients with mild-to-moderate pulmonary cryptococcosis should be treated with fluconazole (400 mg/d) for 6–12 months. Alternative regimens include voriconazole or posaconazole.^144^

Of note, monitoring the titers of cryptococcal antigen is not recommended as it may remain elevated.^74^

#### Aspergillus

Treatment for invasive aspergillosis should be initiated as soon as the diagnosis is suspected. Voriconazole is the treatment of choice based on a randomized control trial that showed improved survival compared with amphotericin B deoxycholate (70.8% vs. 57.9%).^145^ More recently, the SECURE trial demonstrated that isavuconazole was noninferior to voriconazole in treating invasive mold infections. Proven or probable invasive pulmonary aspergillosis represented 83% of all mold cases. Patients treated with isavuconazole had lower hepatobiliary side effects than those treated with voriconazole.^146^ The VITAL trial assessed the use of isavuconazole treatment for rare fungal diseases, including patients with invasive aspergillosis with renal impairment. While it was not compared with voriconazole, the treatment efficacy was similar to historical cohorts.^147^

In another study, posaconazole was shown to be noninferior to voriconazole and can be used as an alternative for primary treatment.^148^ Just like voriconazole, posaconazole requires therapeutic drug monitoring for toxicity. In patients unable to tolerate a triazole medication or have drug interactions that preclude the use of triazoles, liposomal amphotericin B can be used as an alternative, and a study comparing high dose versus standard dose showed similar survival rates of the standard dose arm historically with voriconazole (~70%). Of note, the high dose was not associated with improved outcomes.^149^ Combination therapy of triazoles with echinocandins is reserved for patients with refractory disease. However, a study by Mar et al^150^ suggested a trend toward an improved outcome in combination therapy versus monotherapy with voriconazole. The duration of therapy is usually continued for 6–12 weeks though individual duration depends on the clinical and radiographic response.^84^

#### Pneumocystis

Most of the evidence of the management of pneumocystosis is derived from clinical trials in HIV-infected individuals. Trimethoprim-sulfamethoxazole 15–20 mg/kg of the trimethoprim component divided into 3 or 4 doses over 24 hours for 21 days is the most widely recommended therapeutic regimen. Alternative agents dapsone with trimethoprim, atovaquone, clindamycin with primaquine, or pentamidine.^151^

Adjunctive corticosteroids are recommended for patients with substantial hypoxemia (arterial oxygen partial pressure <70 mm Hg or alveolar-arterial gradient >35 mm Hg on room air) as it decreases the overall mortality and the need for mechanical ventilation.^152^

PCP prophylaxis is recommended in patients who are receiving a glucocorticoid dose equivalent to >20 mg of prednisone daily for over 4 weeks.^97^ Prophylaxis guidelines following transplantation are variable, with some recent studies calling into question the widespread use of PCP prophylaxis among all liver transplant recipients and others suggesting lifelong prevention.^36,153^ The current AST IDCOP guidelines recommend prophylaxis for at least 6–12 months post-transplant for all solid organ transplants except lung and small bowel transplant recipients whom they recommend lifelong prophylaxis. Lifelong secondary prophylaxis is also recommended in solid organ transplant recipients (or in patients with persistent immunosuppression) with a prior episode of PCP.^153^

The agent of choice is trimethoprim-sulfamethoxazole that can be given as 80/400 mg daily or 160/800 mg daily or thrice a week. Alternative regimens include dapsone 50 mg twice daily or 100 mg daily (hemolytic anemia can occur in patients with G6PD deficiency) and atovaquone 1500 mg daily or aerosolized pentamidine 300 mg once a month.^97^

### Dimorphic fungi

#### Histoplasma

Patients with moderately severe to severe acute pulmonary disease should be managed with liposomal amphotericin B 3 mg/kg daily for 1–2 weeks, followed by itraconazole monotherapy (200 mg every 8 h for 3 d followed by 200 mg every 12 h). The length of therapy with itraconazole depends on the patient’s net state of immunosuppression. While immunocompetent patients should receive a minimum of 12 weeks of itraconazole, immunocompromised patients should receive a minimum of 1 year.

Adjuvant corticosteroids (methylprednisolone 0.5–1 mg/kg daily) during the first 1–2 weeks of antifungal therapy are recommended for patients who develop acute hypoxemic respiratory failure.^103,154^

Patients with progressive disseminated disease should be managed with liposomal amphotericin B 3 mg/kg daily for 1 to 2 weeks followed by itraconazole monotherapy (200 mg every 8 h for 3 d followed by 200 mg every 12 h) for at least 12 months regardless of their immune status.^103,154^

Immunocompromised patients with mild pulmonary disease should be treated with itraconazole (200 mg every 8 h for 3 d followed by 200 mg every 12 h) for a minimum of 12 weeks. Immunocompetent patients with mild pulmonary disease usually do not require therapy unless symptoms are present for over 4 weeks.^103,154^

Primary prophylaxis against histoplasmosis is not routinely recommended even in highly endemic areas. However, the patients who have recovered from active histoplasmosis during the 2 years before the initiation of immunosuppression may be considered for secondary azole prophylaxis.^103^

#### Blastomyces

Patients with moderately severe to severe acute pulmonary disease or those with progressive disseminated infections should be managed with liposomal amphotericin B 5 mg/kg daily for 1–2 weeks, followed by itraconazole monotherapy (200 mg every 8 h for 3 days followed by 200 mg every 12 h) for a total of at least 12 months.

Patients presenting with mild pulmonary disease should be treated with itraconazole (200 mg every 8 h for 3 d followed by 200 mg every 12 h) for 6–12 months.^103,155^

#### Coccidioides

Treatment is not routinely recommended for immunocompetent patients with mild-to-moderate primary pulmonary coccidioidomycosis with or without pulmonary nodules and in asymptomatic cavitary disease. However, patients with a debilitating illness or extensive pulmonary involvement should receive antifungal therapy. The treatment of choice is fluconazole 400–1200 mg daily. Itraconazole is an alternative option. While the extent of duration is not certain, most experts recommend that the therapy should be extended for 3–6 months. In contrast to pulmonary manifestations, all patients with extrapulmonary soft tissue coccidioidomycosis should receive therapy for at least 6–12 months.^156^

Patients presenting with meningeal involvement should receive lifelong therapy as azole therapy alone appears to suppress rather than cure coccidioidomycosis meningeal disease. Currently, high-dose fluconazole (800–1200 mg daily) is considered the regimen of choice.^156^ There are no human studies to show that itraconazole or other azoles are superior to fluconazole though a murine model showed modest benefits of itraconazole over fluconazole.^157^

Immunocompromised individuals with acute or chronic pulmonary coccidioidomycosis who are clinically stable should receive fluconazole 400 mg daily for 6–12 months followed by lifelong suppressive therapy with 200–400 mg daily. For patients who present with severe disease, rapidly progressive or disseminated disease, the current guidelines recommend liposomal amphotericin B 5 mg/kg daily until the patient has stabilized followed by fluconazole 400–1200 mg daily for 6–12 months followed by lifelong suppressive therapy with fluconazole 200–400 mg daily.^103,156^

Transplant recipients in endemic areas without active coccidioidomycosis should receive oral azole prophylaxis for a minimum of 6–12 months post-transplant regardless of their pretransplant serostatus.^103^ Some experts recommend lifelong suppressive therapy in lung transplant recipients in the endemic.^103,158^

### Antifungal drug considerations

The use of azole medications (eg, fluconazole, itraconazole, isavuconazole, posaconazole, and voriconazole) is well-known to be associated with hepatic function abnormalities. The range of drug-induced liver injuries may range from mild asymptomatic elevations of liver function tests to severe hepatic dysfunction, including hepatitis, cholestasis, and fulminant hepatic failure. Antifungal-induced hepatic injury is often characterized as an acute, cholestatic, or mixed hepatocellular–cholestatic response.^140,159^

In a UK cohort of 69830 patients requiring antifungal therapy, acute liver injury occurred in 2 patients taking ketoconazole, 2 taking itraconazole, and none taking fluconazole.^160^ In a Taiwanese cohort of 90847 patients taking oral antifungal agents, 28 DILI cases used ketoconazole, 12 fluconazole, and 3 itraconazole.^161^ Of note, neither of these cohorts reported the number of patients with underlying liver disease.

Therapeutic drug monitoring (ie, circulating drug levels) is indicated when the standard dosing of any given drug results in marked variability in drug blood concentrations and when relationships between drug exposure (plasma drug level) and either efficacy or toxicity have been established. The plasma drug levels of multiple azoles vary due to inconsistent absorption, metabolism, elimination, or interaction with concomitant medications.^162–164^

Fluconazole and isavuconazole have predictable (linear) pharmacokinetics. Itraconazole, posaconazole, and voriconazole have variable pharmacokinetics leading to significant variability in their blood concentrations. Hence, therapeutic drug monitoring is recommended for those antifungals.^160,163^ During therapy with azoles, liver functions tests should be monitored periodically.^140^

Echinocandins (eg, anidulafungin, caspofungin, micafungin, and rezafungin) and polyenes (ie, amphotericin B) have seldomly been associated with hepatotoxicity and do not require therapeutic drug monitoring.

### Outcomes

#### Overall mortality

In a large multicenter cohort of over 2700 patients with cirrhosis, fungal infections were associated with increased risk for ACLF, ICU admission, and worse 30-day survival compared with patients without fungal infection.^48^ Overall mortality was 30% for all fungal infections, with over 50% mortality for patients with fungemia and fungal peritonitis.^45^ When looking specifically at patients with ACLF and acute decompensation of liver disease, the 90-day mortality was 71%,^49^ with similarly high mortality rates among other cohorts of patients with ACLF complicated by bacterial and/or fungal infections.^47^

#### Fungal peritonitis and cirrhosis

Patients with fungal peritonitis had a threefold increase in mortality rate compared with patients with a bacterial etiology for their peritonitis. Furthermore, patients with fungal peritonitis have an increased time to diagnosis and delayed use of antifungal agents, highlighting the need to have a high index of suspicion for this illness.^7^ Only 2 patients survived in a small case series of 16 patients with cirrhosis and cryptococcal peritonitis. Interestingly, only a fifth of the patients had a polymorphonuclear cell count of >250 per mm^3^, contributing to a delay in diagnosis, resulting in only seven out of 16 patients receiving antifungal treatment. Unfortunately, many patients are correctly diagnosed only at autopsy.

In a recent review of 87 cases of cryptococcal infection in decompensated cirrhosis, the reported 90-day mortality exceeded 80% compared with 13.6% and 22.7% in the HIV-positive and HIV-negative groups, respectively.^165^ Cryptococcal infection has a 90-day mortality of 14% though it can be higher in patients with disseminated disease and renal failure^166^ but may be worse in patients with liver diseases owing to a delay in diagnosis. In cirrhosis, the risk factors for mortality include a high MELD, a requirement for hemodialysis or mechanical ventilation, hypotension/shock, altered mental status, and fungemia.^165^

Mortality associated with fungal infections in AH has been reported to range from 33% to 66%.^167^ In the STOPATH study, prednisolone was associated with a reduction in 28-day mortality though it was associated with an increased number of serious infections.^168^

The improvement in surgical technique, prevention, and medical management has decreased the incidence and mortality of fungal infections in liver transplant recipients. However, IFIs continue to carry high morbidity and mortality. Invasive candidiasis carries a 90-day mortality of 21%–34%. This varies depending on the species and comorbidities.^169–171^ The mortality is higher for nonalbicans species (27%–52%) than *C. albicans* at 22%.^172^

#### Other fungi

Due to their invasive and necrotizing pathogenesis, infections due to molds carry higher mortality. In liver transplant recipients, invasive aspergillosis has an overall mortality of 66%.^173^ In AH, invasive aspergillosis carries a grim prognosis with 0% 3-month transplant-free survival compared with 53% of patients without invasive aspergillosis.^12^ Other studies have also found a higher rate of IFI among patients who receive corticosteroids for the treatment of AH than those who did not, with high mortality rates among all patients but highest among those who had received corticosteroids.^167^ However, it is important to consider that steroids may indicate more severe liver disease and dysfunction, complicating the interpretation of these findings.

For endemic fungi, the TRANSNET study showed that the mortality of endemic mycoses was 16% in a transplant cohort.^174^ The mortality was lower in histoplasmosis (10%) than in blastomycosis (21%) or coccidioidomycosis (30%–50%).^175–177^

### Future directions

IFIs continue to have high morbidity and mortality in patients with liver disease. Patients with cirrhosis, severe AH, and liver transplant recipients are all at increased risk of acquiring IFI with poor outcomes. While advances have been made in diagnostic techniques, more accurate rapid tests are still required. Host immune susceptibility profiling, including neutrophil function activity, may allow for preemptive prophylactic strategies in selected patients at elevated risk for fungal infections.^18,51^ A high index of suspicion in at-risk patients is required for timely diagnoses and early therapeutic intervention. In addition, high-risk post-liver transplant patients benefit from antifungal prophylaxis. Complicating matters further, shifting epidemiology, and increasing resistance to current antifungal therapies pose a major challenge to current prophylaxis and empiric regimens. Host susceptibility profile and microbiome dysbiosis analysis may be useful strategies to stratify patients and define better preventive strategies.
